# Application of optical coherence tomography in multiple sclerosis: consensus recommendations of the Austrian network (AN-OCT-MS)

**DOI:** 10.1007/s00415-025-13537-8

**Published:** 2025-12-13

**Authors:** Gabriel Bsteh, Luisa Velez Escola, Harald Hegen, Ewald Lindner, Michael Khalil, Sophie Schneider, Gerhard Traxler, Erich Raithel, Christian Bsteh, Christoph Mitsch, Thomas Berger, Berthold Pemp

**Affiliations:** 1 Department of Neurology, Medical University of Vienna, Waehringer Guertel 18-20, 1090 Vienna, Austria; 2 Comprehensive Center for Clinical Neurosciences and Mental Health, Medical University of Vienna, Vienna, Austria; 3 Department of Ophthalmology, Medical University of Innsbruck, Innsbruck, Austria; 4 Department of Neurology, Medical University of Innsbruck, Innsbruck, Austria; 5 Department of Ophthalmology, Medical University of Graz, Graz, Austria; 6 Department of Neurology, Medical University of Graz, Graz, Austria; 7 Department of Ophthalmology, Med Campus III, Kepler University Hospital GmbH, Linz, Austria; 8 Department of Neurology 2, Med Campus III, Kepler University Hospital GmbH, Linz, Austria; 9 Radstadt, Austria; 10 Salzburg, Austria; 11 Department of Ophthalmology, Klinik Donaustadt, Vienna, Austria; 12 Department of Ophthalmology, Medical University of Vienna, Vienna, Austria

**Keywords:** Multiple sclerosis, Optical coherence tomography, Consensus

## Abstract

Optical coherence tomography (OCT) for measuring retinal layer thicknesses is a new modality relevant for the diagnosis and prognosis of multiple sclerosis (MS), which is now anchored in clinical routine at the latest with the 2024 revision of the McDonald criteria. The aim of this consensus paper, prepared by the members of the Austrian Network for Optical Coherence Tomography in Multiple Sclerosis (AN-OCT-MS), is to provide a acomprehensive recommendation for the use of OCT in patients with MS.

**Supplementary Information:**

The online version contains supplementary material available at 10.1007/s00415-025-13537-8.

## Introduction

The diagnosis, treatment, and monitoring of patients with multiple sclerosis (MS) are characterized by a high degree of complexity, wherefore their management should be carried by neurologists specializing in MS care in accordance with international consensus guidelines [1, 2].

Optical coherence tomography (OCT) for measuring retinal layer thicknesses is a new modality relevant for diagnosis and prognosis, which is now anchored in clinical routine with the 2024 revision of the McDonald criteria [3, 4].

The aim of this consensus paper prepared by the members of the Austrian Network for Optical Coherence Tomography in Multiple Sclerosis (AN-OCT-MS) is to provide a comprehensive recommendation for the use of OCT in patients with MS.

## Methods

This document has been developed under the auspices of the Austrian Society of Neurology (ÖGN) and the Austrian Society of Ophthalmology (ÖOG), following a formal consensus methodology [5]. It covers indications as well as quality standards for conduction, interpretation, and reporting of OCT in patients with MS.

An expert committee was set up, comprising neurologists with specific expertise in the management of MS and (neuro)ophthalmologists with specific expertise in OCT. The committee identified the scope and topics formulating clinical questions. The formulation and agreement of the recommendations were done using the modified Nominal Group Technique, which is a highly structured procedure, based on iterative ratings with feedback, to reach consensus in a small group of experts on topics for which expert opinion is relevant [5]. The evidence was presented and discussed within the expert committee members and other invited discussants during a focused workshop held in Vienna in February 2024.

As a result, the first set of statements was circulated to the core working group members for a first round of voting through email, using a percent agreement scale, with a pre-defined 80% level of agreement. The revised statements/recommendations were submitted for agreement in a further round of voting through email. Before submission in October 2025, the literature was critically reviewed and updated where deemed necessary.

The definitiveness of each statements/recommendation reflects the grade of agreement reached during the consensus process, rather than the strength of empirical evidence, and should therefore be interpreted as consensus-based guidance unless otherwise supported by data or references. The manuscript was reviewed and approved by all members of the expert committee and endorsed by the management boards of the ÖGN and ÖOG.

## Results and recommendations

### Indications for OCT in MS

The measurement of retinal layer thicknesses using OCT is a surrogate marker of MS-associated neuroaxonal damage. This can be caused either directly as a result of optic neuritis (ON) or indirectly by retrograde transsynaptic degeneration secondary to damage to cerebral structures. The relevant parameters are the thickness of the peripapillary retinal nerve fiber layer (pRNFL), which represents the total number of unmyelinated axons of the optic nerve, and the thickness of the macular ganglion cell and inner plexiform layer (mGCIPL), which contains the neuronal cell bodies of the optic nerve and is sometimes visualized only as the macular ganglion cell layer (GCL). OCT of the retina is non-invasive (painless, no risk of damage, no side effects), fast, cost-effective, and can be repeated as often as required.

The AN-OCT-MS agreed on four indications in which OCT can be used in patients with MS (see Table 1).

**Table 1:** Overview of indications for OCT in MS

Indication	Time point	Aim	Finding	Interpretation
1) Diagnosis	Investigation of suspected MS	Evidence of affection of the optic nerve to establish dissemination in space (DIS) according to McDonald criteria 2024 (5th region)	Inter-eye difference	Evidence of optic nerve affection is considered as established if relevant differential diagnoses have been excluded
			pRNFL ≥ 6 μm/≥ 6% and/or GCIPL ≥ 4 μm/≥ 4%	
2) Prognosis	Initial diagnosis/first contact^#^	Stratification of risk of physical and cognitive disability progression	pRNFL ≤ 88 μm^*^	Signs of advanced MS-associated neuroaxonal degeneration
	Starting/changing therapy^#^	(surrogate marker for neuroaxonal reserve)	GCIPL < 77 μm^*^	(two to three times increased risk of disability progression in the next 3 years)
3) Optic neuritis	At least ≥ 3 months after ON symptom onset	Quantification of the neuroaxonal damage caused by ON	Layer thickness loss in the affected eye^**^	Severe ON with increased risk of incomplete remission of visual symptoms
			pRNFL loss > 20 µm and/or GCIPL loss > 10 µm	(potentially also increased risk of incomplete remission of future relapses)
*4) Treatment monitoring*	*Rebaselining at least* ≥ *3 months after DMT start*^*#*^	*Quantification of MS-associated neuroaxonal damage*	*Layer thickness loss*	*Interpretation currently only within the overall clinical context and at centers particularly familiar with the application*
	*Repeat every 12–24 months*^*#*^		*pRNFL and/or GCIPL*	

ON: optic neuritis. pRNFL: peripapillary retinal nerve fiber layer. GCIPL: ganglion cell and inner plexiform layer

^*^For patients with no history of ON and no pathological inter-eye difference (pRNFL ≥ 6 µm/≥ 6% and/or GCIPL ≥ 4 µm/≥ 4%), the mean value of both eyes is used; for patients with a history of unilateral ON and/or pathological IED, the value of the better side is used; for patients with a history of ON in both eyes, the stratification is not validated

^**^Comparison with last available OCT according to AN-OCT-MS protocol

^#^at least ≥ 3 months after the last ON

#### Indication 1: Evidence of optic nerve involvement as a supporting feature for the diagnosis of MS

Diagnosis of MS requires evidence of dissemination in space (DIS) and time (DIT). The continuous development of diagnostic criteria for MS through the inclusion of paraclinical examinations such as magnetic resonance imaging (MRI) and cerebrospinal fluid analysis has led to a faster and more accurate diagnosis and paved the way for earlier access to disease-modifying interval therapy (DMT) for MS patients [6–8]. The optic nerve is a typical manifestation site in MS. ON is the initial clinical manifestation in approximately 25% of MS patients, with a further 10–30% of patients with other initial manifestations showing signs of asymptomatic optic nerve involvement at diagnosis [9–12]. However, as the optic nerve was not considered a region for the detection of DIS in 2017 McDonald criteria, ON was less likely to lead to an MS diagnosis than a symptomatic lesion of the brainstem or spinal cord [13]. As an illustrative example, a patient with a symptomatic spinal cord lesion and a contrast-enhancing periventricular lesion on MRI could have been diagnosed with MS, whereas an identical patient with ON instead of a symptomatic spinal cord lesion could not have been diagnosed with MS based on the previous criteria. ON leads to neuroaxonal damage to the optic nerve, which can be measured by OCT as reduced thickness of pRNFL and/or mGCIPL [14, 15]. The inter-eye difference (IED) in pRNFL and or mGCIPL thickness has been shown to be a sensitive and robust marker of ON in several methodologically valid and independently performed studies [12, 15–23]. IED thresholds of ≥ 4 µm and/or ≥ 4% in the mGCIPL and/or ≥ 5 µm and/or ≥ 5% in the pRNFL identify both symptomatic and asymptomatic lesions and are longitudinally stable, i.e., even lesions dating back years can be identified. Several retrospective and prospective studies have shown that the addition of the optic nerve as a fifth region increases the sensitivity for the detection of DIS without compromising specificity [24–26]. Therefore, the optic nerve is included as a fifth DIS region in the 2024 revision of the diagnostic criteria according to McDonald, whereby affection of at least two regions constitutes detection of DIS, with additional scenarios in case of only one affected region [3]. However, it must be taken into account that an abnormal IED on OCT is not specific for MS and can also occur due to other diseases of the optic nerve (e.g., glaucomatous, ischemic or compressive optic neuropathy, optic disc drusen) or the retina (e.g., retinal vascular occlusion, diabetic retinopathy, maculopathies). By definition, application of the 2024 McDonald criteria a priori requires MS to be the most likely diagnosis and, thus, the exclusion of relevant plausible alternative diagnoses [3]. As pRNFL has a slightly higher margin of measurement error, the IED threshold for pRNFL in the 2024 McDonald criteria was set at ≥ 6 µm and/or ≥ 6% (instead of the ≥ 5 µm and/or ≥ 5% used in most studies) to lower the risk of misclassification [3, 4]. Based on this evidence, the AN-OCT-MS recommends performing an OCT as part of the diagnostic workup for suspected MS. If a pathological IED of the pRNFL (IED ≥ 6 µm/≥ 6%) and/or the mGCIPL (IED ≥ 4 µm/≥ 4%) is detected, an affection of the optic nerve can be considered proven for the detection of DIS, provided that other causes of a pathological IED have been excluded.

***3.1.2 Indication 2: Stratification of global neuroaxonal damage and risk of physical and cognitive disability progression at diagnosis or initiation/change of disease-modifying treatment.***

MS is characterized by a largely overlapping continuum of acute focal demyelinating inflammation and progressively accumulating neuroaxonal damage [27]. The latter typically remains clinically ineloquent in the early phase of the disease due to the presence of neuroaxonal reserve, but is a major determinant of long-term prognosis [27, 28]. Detection of neuroaxonal damage, i.e., a proxy of the remaining neuroaxonal reserve, in the early stages of MS is therefore an essential tool for prognostic stratification and the choice of treatment strategy [27].

Numerous studies and a systematic meta-analysis show that the thinning of pRNFL and mGCIPL is significantly more pronounced in patients with advanced MS and correlates with longer disease duration, higher EDSS, increased cognitive impairment, and more pronounced global and focal brain atrophy [15]. Therefore, pRNFL and mGCIPL thickness are considered surrogate markers of the extent of neuroaxonal loss that has occurred in the global central nervous system and, thus, of the remaining neuroaxonal reserve. Several studies have independently shown that MS patients with a pRNFL thickness ≤ 88 µm or an mGCIPL thickness < 77 µm have a two- to threefold increased risk of progression of physical or cognitive disability within the next 3 years, regardless of the time of measurement [29–32]. About one-third of patients undercut these thresholds, respectively. Importantly, ON history must be taken into account when applying these thresholds. For patients without a history of ON, the mean values of both eyes are used; for patients with prior unilateral ON or pathological IED (pRNFL ≥ 6 µm/≥ 6% and/or mGCIPL ≥ 4 µm/≥ 4%), only the values of the better side are used [29, 30, 32]. For patients with a history of ON in both eyes, however, the described stratification has not been validated.

OCT may also be helpful as a decision criterion before starting/escalating DMT, although no formal intervention study is available to date [33].

Based on this evidence, the AN-OCT-MS recommends performing OCT for prognostic stratification at diagnosis/first contact or prior to DMT initiation/switch. If there are signs of advanced neuroaxonal damage (pRNFL ≤ 88 µm or mGCIPL < 77 µm), a two- to threefold increased risk of disability progression over the next 3 years can be assumed.

#### Indication 3: Quantification of neuroaxonal damage after ON

The extent of neuroaxonal damage to the optic nerve as a result of ON can vary greatly depending on the severity of ON, the timing and extent of acute therapy, DMT, and the degree of remyelination [9, 34]. OCT can be used to quantify this neuroaxonal damage. Depending on the severity of ON, a range of thinning of the pRNFL from 6 µm to > 30 µm and of the mGCIPL from 4 µm to > 20 µm can be expected compared to the contralateral eye or to any previous findings [14, 34]. The timing of examination is crucial here: while in the acute phase of MS-associated ON usually no change or even swelling of the pRNFL is observed compared to the contralateral eye, the extent of pRNFL/mGCIPL thinning as an expression of neuroaxonal damage to the optic nerve only becomes detectable 3e months after ON [14, 34]. OCT in this indication should therefore be performed at the earliest 3 months after the onset of ON. The degree of pRNFL/mGCIPL loss correlates strongly with the impairment of visual function (visual acuity, contrast vision) that remains residual after ON [9, 14, 34]. One retrospective study reported that the extent of pRNFL/mGCIPL thinning is also associated with the risk of incomplete remission of future relapses after the occurrence of ON, independent of other influencing variables [35]. Based on the available evidence, the AN-OCT-MS recommends performing an OCT at least 3 months after each ON to quantify the neuroaxonal damage caused by the ON. If a pRNFL loss of > 20 µm or an mGCIPL loss of > 10 µm is detected in the affected eye, a significant increase in the risk of incomplete recovery from ON and possibly also from case of future relapses can be assumed.

#### Indication 4: Quantification of longitudinal accumulation of neuroaxonal damage

Reliably quantifying neuroaxonal damage during disease course and under DMT remains one of the greatest challenges in monitoring MS [27].

Several methodologically valid retrospective and prospective studies show that pRNFL and mGCIPL atrophy is more pronounced in patients with relapses, physical or cognitive disability progression, progression independent of relapses (PIRA), and progression independent of relapses and MRI activity (PIRMA), and rapid brain atrophy, but is reduced under highly effective DMT [36–48]. Therefore, pRNFL and mGCIPL atrophy are also considered surrogate markers for monitoring MS-associated neuroaxonal damage in the global central nervous system and, thus, also for therapy monitoring.

However, it should be noted that the absolute effect size (i.e., the differences in the extent of retinal atrophy between healthy controls, stable and active MS patients) over a period of 12–24 months is small, so that although a distinction is significant at the group level, it is currently not sufficiently reliable at the individual level. The interpretation of retinal atrophy should therefore be made with great caution. With regard to therapy monitoring, it should also be noted that pRNFL and mGCIPL thickness reduction is primarily caused by retrograde (descending) axonal degeneration, which is why a therapeutic effect of DMT only becomes measurable with a latency of 6–12 months [49–51]. Therefore, the accuracy of pRNFL and mGCIPL atrophy in DMT monitoring is likely to be improved by performing a rebaselining OCT 3–6 months after the start of therapy [52].

Based on the currently available evidence, the AN-OCT-MS cannot generally recommend the use of OCT in treatment monitoring. However, OCT can be used to quantify MS-associated neuroaxonal damage at centers that are particularly familiar with its use, although the interpretation should currently only be made in the overall clinical context and should never be used as the sole decision-making criterion.

### Quality standard for referral to OCT in MS

MS patients should only be referred to an OCT in the context of MS by neurologists or centers with an MS specialization. To ensure high-quality reports and to derive the maximum benefit from the examination, a precise referral is necessary, which should contain at least the following essential information:

- Diagnosis (suspected MS/known MS with details of the course of the disease).
- Clinical evidence of acute or past optic neuritis including which side was affected.
- Indication (OCT according to AN-OCT-MS protocol in indication 1/2/3/4).
- Known other current/previous eye disease(s).

To ensure quality and avoid misconceptions, each MS center should create a defined local or regional network in interdisciplinary exchange with intra- or extramural ophthalmologists, taking into account the personnel and local conditions, to make OCT according to the AN-OCT-MS protocol accessible to all MS patients.

### Quality standard for implementation

Technically speaking, OCT is an imaging procedure that uses low-coherence laser light in the near-infrared range to produce high-resolution cross-sectional images of tissues such as the retina and the bulbar optic nerve in vivo. The OCT scan can be performed by methodically experienced physicians or appropriately trained medical-technical staff. It should be noted that careful training, instruction, and supervision of the medical-technical staff is required if the OCT scan is not performed by the diagnostic physicians themselves. In the event of a spatial and/or personnel separation of the individual service steps (performance of the OCT scan versus diagnosis), the medical technicians must ensure sufficient quality of the images in accordance with established quality criteria (OSCAR-IB) [53].

#### Use of different OCT devices

Device platforms from different manufacturers are available for generating OCT scans, but they do not follow uniform standards. One major difference concerns the acquisition technique using spectral domain OCT (SD-OCT) and swept-source OCT (SS-OCT). While SS-OCT has a faster image acquisition rate and therefore advantages in the acquisition of dynamic processes, SD-OCT offers a higher spatial resolution of the inner retinal layers including pRNFL and mGCIPL. SD-OCT is therefore superior for the purposes of OCT in MS. However, there are also differences between the various SD-OCT platforms, for example for the precise definition of anatomical boundaries for automatic slice thickness determination or scaling with regard to the specified values in the metric system. Therefore, the absolute slice thickness values are not directly comparable between the different device platforms [54, 55]. More than 90% of studies underlying these recommendations were performed on the SD-OCT device platforms Spectralis® (Heidelberg Engineering) and Cirrus® (Carl Zeiss Meditec) [15]. Although these show excellent agreement at the group level, they can diverge significantly at the individual level [54–57]. Both device platforms can therefore be used in all the indications described, but they are not interchangeable within individuals, i.e., a patient should always be examined with the same device platform. The consensus panel found that there is currently no sufficient validation for other device platforms (e.g., Topcon or Nidek). The AN-OCT-MS therefore recommends the use of the Spectralis® or Cirrus® device platforms for OCT scans in MS patients.

#### OCT scan protocol

A peripapillary ring scan and a multilinear macular scan are required to acquire the layer thicknesses of pRNFL and mGCIPL that are relevant for MS applications. The large number of variable scan parameters poses a challenge. The use of a standardized scan protocol is therefore essential (for details see Table 2). In principle, the scans can be performed in a darkened room without pupil dilation. Pharmacological pupil dilation is only necessary in individual cases where the scan quality is otherwise insufficient. For the pRNFL measurement, the standard ring scan should be used with an automatic real-time tracking [ART] of at least 20 and ideally 100 averaged images. For mGCIPL measurement, a multilinear macular volume scan centered on the fovea and an ART of at least 9 and ideally 49 should be performed [48]. On Spectralis®, the thicknesses of the GCL and IPL must be added manually to determine the mGCIPL thickness (see Supplemental Box 1). The AN-OCT-MS recommends the use of the AN-OCT-MS protocol for OCT scans in MS patients.

**Table 2:** OCT scan protocol according to AN-OCT-MS

	**Spectralis® (Heidelberg Engineering)**	**Cirrus® (Carl Zeiss Meditec)**
pRNFL	Peripapillary ring scan (12°): RNFL-N-order RNFL-Scan ART 100 Frames^#^	Peripapillary ring scan (3,46 mm): Optic disc cube 200 × 200
GCIPL^*^	Multilinear macular scan: PPoleH with APS: 30° × 25°^##^	Multilinear macular scan (6 × 6 mm): Macular cube 512 × 128 or Macular cube 200 × 200
Follow-up scans	Follow-up after creating a reference scan	Track to prior

^*^If GCIPL output is not possible for administrative reasons, alternatively specify the GCL in µm or mm^3^

^#^At least ART 20

^##^Alternatively 20° × 20° macular scan, 61 lines (at least 25 lines), ART 49 frames (at least ART 9)

#### Scan quality control

Sufficient quality of the OCT scans is essential for the use of OCT in the indications described. However, there are numerous possibilities for measurement errors due to artifacts or inadequate performance of the examination. All relevant studies on the use of OCT in MS refer to OCT scans that have undergone quality control in accordance with the OSCAR-IB quality control criteria (see Tables 3 and 4) (Table 5) [53].

**Table 3:** OSCAR-IB quality control criteria for retinal OCT scans (modified according to Tewarie et al. [53]**)**

	**Criterion**
**O**	**O**bvious problems not covered by the following items
**S**	Is the OCT **s**ignal sufficient?
	Spectralis: signal strength > 15 (ring- and volume scans) with appropriate averaging of multiple scans (ART activated)
	Cirrus: signal strength > 5
**C**	Is the ring scan correctly centered?^*^
	For circular discs: optic nerve head (ONH) must not cross 4° und 8° radii
**A**	Is there an **a**lgorithm failure?
	Ring scan: segmentation lines correctly identify the superior and inferior RNFL borders
	Macular scan: segmentation lines correctly identify the retinal borders
**R**	Is there a visible **r**etinal pathology, which may potentially impair the parameter reading? See Table 4
**I**	Is the fundus well **i**lluminated?
	Retinal structures visible (ring and macular scans)
**B**	Is the measurement **b**eam placed centrally?
	Homogenous outer ONL reflexion (ring and macular scans)

^*^With Spectralis® readjustment is possible for the macular scan, but not for the ring scan. If ring scan is not centered correctly, scan needs to be repeated

**Table 4:** Overview of relevant retinal pathologies

Category	Pathologies
Structural	Drusen, cysts, detachment, large discs, small crowded discs, fibrae medullares, nevus, tumor, peripapillary atrophy, papilledema, myopia, or hyperopia > 6 dpt
Vascular	AION, PION, retinal artery occlusion, AVM, cotton-wool spots, ischemia affecting the optic pathways
Autoimmune	NMOSD, MOGAD, paraneoplastic (MAR, CAR), SLE, uveitis, birdshot retinochoroiditis
Infectious	Viral, bacterial, fungal, HIV, Lye, secondary syphilis
Hereditary	LHON, DOA, albinismus, cone dystrophy, retinitis pigmentosa
Iatrogenous	Retina surgery, photocoagulation, solar retinopathy, central serous chorioretinopathy, Purtscher ‘s retinopathy, optic nerve sheath fenestration, brain surgery affecting the optic pathways
Metabolic/toxic	Diabetes, vitamin A deficit, alcohol-, tobacco- and malnutrition-induced amblyopia, amiodarone, chloroquine, vigabatrin
Other	Glaucoma, macular degeneration, acute posterior multifocal placoid pigment epitheliopathy, acute macular neuroretinopathy

**Table 5:** Report structure for OCT according to AN-OCT-MS consensus recommendation

Patient identification	
Indication	Indication 1, 2, 3, and/or 4
OCT device platform used	Spectralis®/Cirrus®
OSCAR-IB criteria	Fulfilled/assessable to a limited extent/not assessable
OCT scans	Peripapillary ring scan bilateral/right/left performed
	Multilinear macular scan bilateral/right/left performed
pRNFL	Right: (µm) Left: (µm)
mGCIPL^*^	Right: (µm) Left: (µm)
Signs of relevant pathology other than MS?^**^	Yes/no
	If yes: which?
Signs of past optic neuritis?	Yes (IED pRNFL ≥ 6 µm/6% and/or mGCIPL ≥ 4 µm/4%)/no
Signs of advanced MS-associated neuroaxonal degeneration?	Yes (pRNFL ≤ 88 µm and/or mGCIPL < 77 µm)^***^/no
In case of referral after optic neuritis (indication 3)	Extent of neuroaxonal loss in the affected eye compared to previous findings: pRNFL and mGCIPL (µm)^*^

^*^If mGCIPL output is not possible for administrative reasons, alternatively output the GCL in µm or mm^3^

^**^see Table 4

^***^For patients without signs of past ON, the mean value of the layer thicknesses of both eyes is used; for patients with unilateral ON and/or pathological IED, the layer thickness of the better side is used; for patients with a history of ON in both eyes, the stratification is not validated

The AN-OCT-MS therefore recommends the application of the OSCAR-IB criteria for quality control for all indications described. Only OCT scans that meet the OSCAR-IB quality criteria should be used for diagnosis.

#### Follow-up scans

Due to the different absolute values on different device platforms, the same patient should always be examined with the same device platform. To avoid possible errors or inaccuracies when comparing OCT scans longitudinally, identical scan parameters as well as optimal alignment of the scan camera and optimal positioning of the head should be ensured. Algorithms for the automatic registration of subsequent to previous OCT scans enable a more precise observation of progression than the analysis of images taken independently of each other. The "Follow-up" or "Track to Prior" functions available on the Spectralis® or Cirrus® device platforms enable individual B-scans or scan fields to be recorded at the most identical location possible at different points in time.

The AN-OCT-MS therefore recommends using the same device (or in the same device network if data is available across locations or devices) and activating the follow-up function for follow-up examinations.

### Quality standard for OCT report

In addition to ensuring technical quality, the quality of the evaluation and assessment of the OCT findings plays a particularly important role. In particular, qualification in the assessment of the ocular fundus is a prerequisite for detecting and correctly classifying relevant changes in OCT imaging. The interpretation of an OCT according to AN-OCT-MS protocol should therefore only be carried out by physicians, primarily (neuro)ophthalmologists or neurologists, who are familiar with the assessment of the fundus and trained in OCT imaging.

The report of an OCT according to AN-OCT-MS protocol should meet the quality criteria of validity, reliability, reproducibility, completeness, comprehensibility, comparability, objectivity, and usefulness.

In any case, the report should indicate which device was used to acquire the OCT scans and whether the OCT scans meet the OSCAR-IB quality criteria.

In accordance with the quality criteria, the OCT scan on which the findings are based should be attached to the findings. Possible formats for this are a paper printout, a PDF file, or a DICOM file.

To ensure comprehensibility and usefulness, the measured variables relevant for the assessment (global layer thicknesses of pRNFL and mGCIPL in the right and left eye) should be explicitly and clearly highlighted in the report and given as numerical values.

Any relevant abnormalities in the OCT scans (e.g., focal atrophy) should be explicitly pointed out.

In addition to the scan, the findings of an OCT according to the AN-OCT-MS protocol should contain a summarized interpretation that explicitly includes the following points:

- Are there signs of a secondary pathology independent of MS that affects the interpretation with regard to indications 1–4?
- Are there signs of optic neuritis according to indication 1?
- Are there signs of advanced MS-associated neuroaxonal damage according to indication 2?
- In case of referral after optic neuritis (indication 3): what is the extent of the neuroaxonal loss in the affected eye (including indication of the loss in pRNFL and mGCIPL in µm)?

### Perspectives

OCT-based retinal layer thickness measurement has now arrived in clinical routine as an additional modality for the diagnosis and prognosis of MS. The AN-OCT-MS consensus represents the first nation-wide consensus, which should inspire others to follow suit in providing patients with MS with comprehensive access to high-quality OCT.

**Fig. 1:**
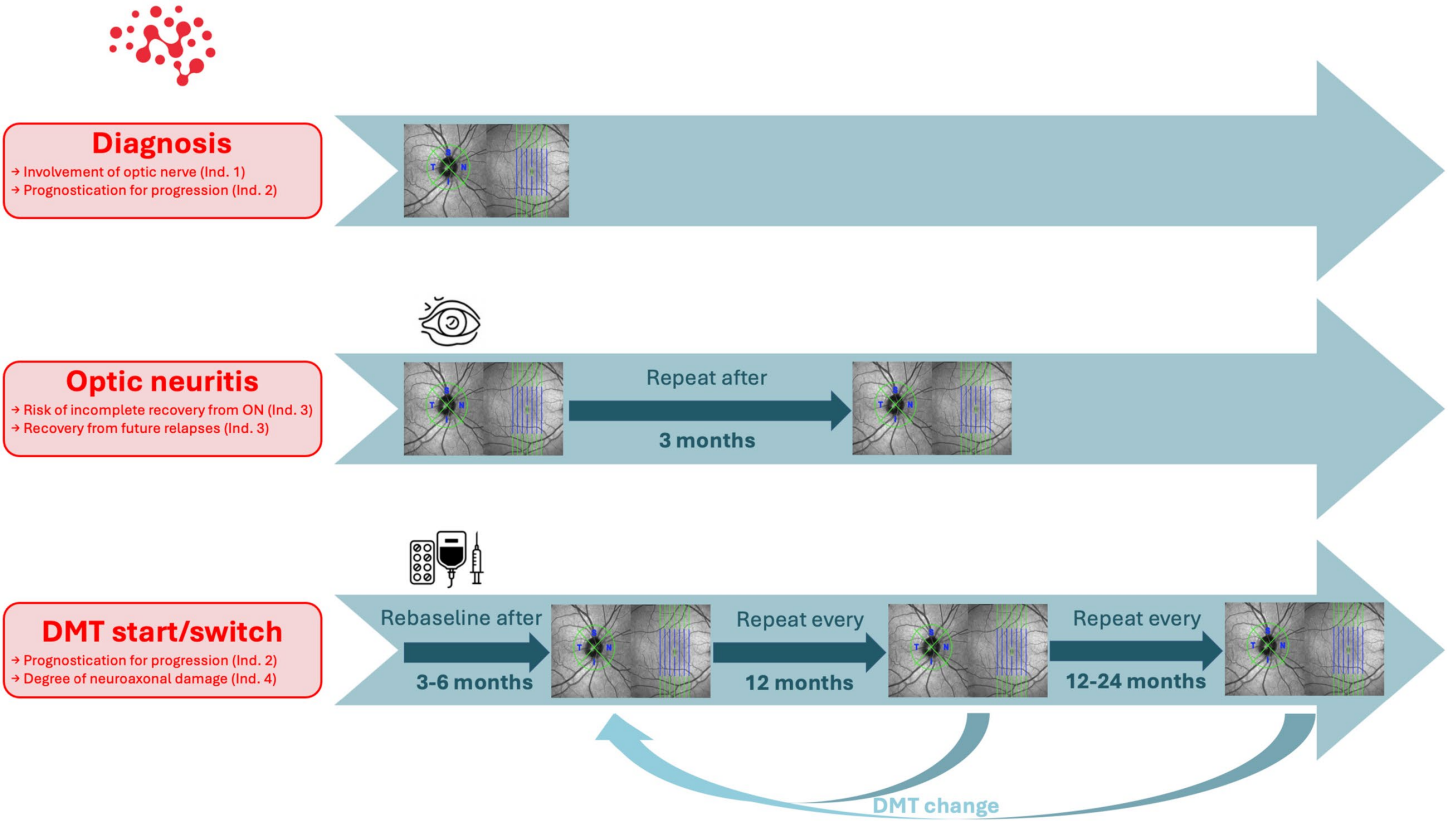
Recommendation for the use of optical coherence tomography in multiple sclerosis. DMT: disease-modifying therapy

Standardized OCT is recommended to detect optic nerve involvement in suspected MS (Indication 1), to support prognostic stratification at diagnosis or before DMT initiation or switch (Indication 2), and 3 months after ON to quantify neuroaxonal damage (Indication 3). In experienced centers, OCT may additionally be used for longitudinal assessment of MS-associated neuroaxonal damage (Indication 4), provided results are interpreted within the clinical context (Fig. 1).

In the sense of a "living" consensus recommendation, it will be re-evaluated annually by the AN-OCT-MS and updated if necessary. On this basis, continuous further training and networking of intra- and extramural neurologists and ophthalmologists is essential. This is to be facilitated by the AN-OCT-MS in a series of national and regional events.

To facilitate access, AN-OCT-MS provides a list of intramural and extramural locations where OCT is offered in accordance with the present consensus recommendation. This also aids in identifying gaps in care and addressing them accordingly.

It is also essential to clarify the current differences in the way intramural and extramural OCT for MS patients is billed in Austria and abroad. This consensus recommendation provides an essential tool for interaction with the interest groups and representatives involved.

However, several limitations should be acknowledged. Although following a structured approach with formal consensus methodology, the AN-OCT-MS recommendation needs to be considered as an expert-based consensus recommendation and, thus, reflects the collective interpretation and clinical experience of specialists rather than the outcome of systematic, evidence-graded guidelines. Consequently, several recommendations rely on expert opinion, particularly in areas where high-quality data are lacking or emerging. While this approach enables timely guidance in a rapidly evolving field, it may introduce subjective bias and variability related to local practice patterns. Furthermore, the consensus reflects the Austrian healthcare context, and its generalizability to other regions may be limited by differences in OCT availability, infrastructure, and reimbursement. The recommendations should therefore be interpreted as a foundation for standardized practice and future research, rather than prescriptive rules, and updated as new evidence and technologies become available.

During the development of this consensus recommendation, relevant gaps in the evidence for the use of OCT in MS were also identified. Future research and international collaboration will be essential to validate these recommendations and to adapt them to broader clinical and geographical contexts. From a pathobiological perspective, future studies should further integrate structural OCT findings with the underlying biological mechanisms of neurodegeneration in MS, including the processes of trans-synaptic axonal degeneration—both anterograde and retrograde—that may link focal lesions to widespread retinal and cerebral damage as well as the role of retinal microvasculature by incorporating OCT angiography methods. From a methodological point of view, the current lack of data on the comparability or convertibility of measured values between the different device platforms as well as the validation of other frequently used devices is particularly important.

From a clinical perspective, there is a clear need for further evidence for the clinical significance of OCT in indications 2–4. The following questions are particularly relevant from the perspective of the AN-OCT-MS:

- Does the use of OCT in patients with MS as a surrogate marker of neuroaxonal reserve (indication 2) have predictive value for treatment response in addition to prognostic value, i.e., specifically: do MS patients with OCT-detected signs of MS-associated advanced neuroaxonal degeneration benefit from more aggressive therapy?
- Does the use of OCT in patients with MS as a surrogate marker of neuroaxonal damage after optic neuritis (indication 3) have predictive value for treatment response in addition to prognostic value, i.e., specifically: do MS patients with OCT-detected pronounced neuroaxonal damage after optic neuritis benefit from more aggressive treatment?
- Is OCT as a surrogate marker of neuroaxonal damage in the longitudinal section (indication 4) capable of validly and reliably detecting subclinical neuroaxonal damage in the longitudinal section at the individual level?
- Can valid and reliable thresholds be established that predict future clinical events (especially disability progression)?
- Does the neuroaxonal damage quantified by OCT in indication 4 progress longitudinally in parallel with the brain atrophy detected by MRI? Are these processes driven by the same pathophysiological mechanisms?
- Is OCT in indication 4 able to validly and reliably detect differences in the extent of subclinical neuroaxonal damage between current and future therapies, i.e., can OCT be used as a marker of treatment response?

Finally, the establishment of this nation-wide consensus places the AN-OCT-MS Network in Austria in an excellent position to generate large-scale, high-quality datasets suitable for future artificial intelligence applications.

## Supplementary Information

Below is the link to the electronic supplementary material.

Supplementary file1 (DOCX 236 KB)

Supplementary file2 (DOCX 15 KB)

Supplementary file3 (DOCX 17 KB)
