# Supplementary material for: Application of optical coherence tomography in multiple sclerosis: consensus recommendations of the Austrian network (AN-OCT-MS)
Source: J Neurol. 2025 Dec 13;273(1):24. doi: 10.1007/s00415-025-13537-8 (PMC12701873; doi:10.1007/s00415-025-13537-8)
Supplement: Supplementary file 1 — Supplementary file1 (DOCX 236 KB) [file 415_2025_13537_MOESM1_ESM.docx]

**Box 1: Formula for the area-corrected calculation of the mean GCIPL thickness from Spectralis® findings from macula scans.**

| **Average GCL thickness:**  ***average thickness inner ring (avT_GCL_IR)*** = (IR_GCL_sup + IR_GCL_nas + IR_GCL_inf + IR_GCL_temp)/4  ***average thickness outer ring (avT_GCL_OR)*** = (OR_GCL_sup + OR_GCL_nas + OR_GCL_inf + OR_GCL_temp)/4  ***avT_GCL_IR+OR = (avT_GCL_IR*2+avT_GCL_OR*6,75)/8,75*** | 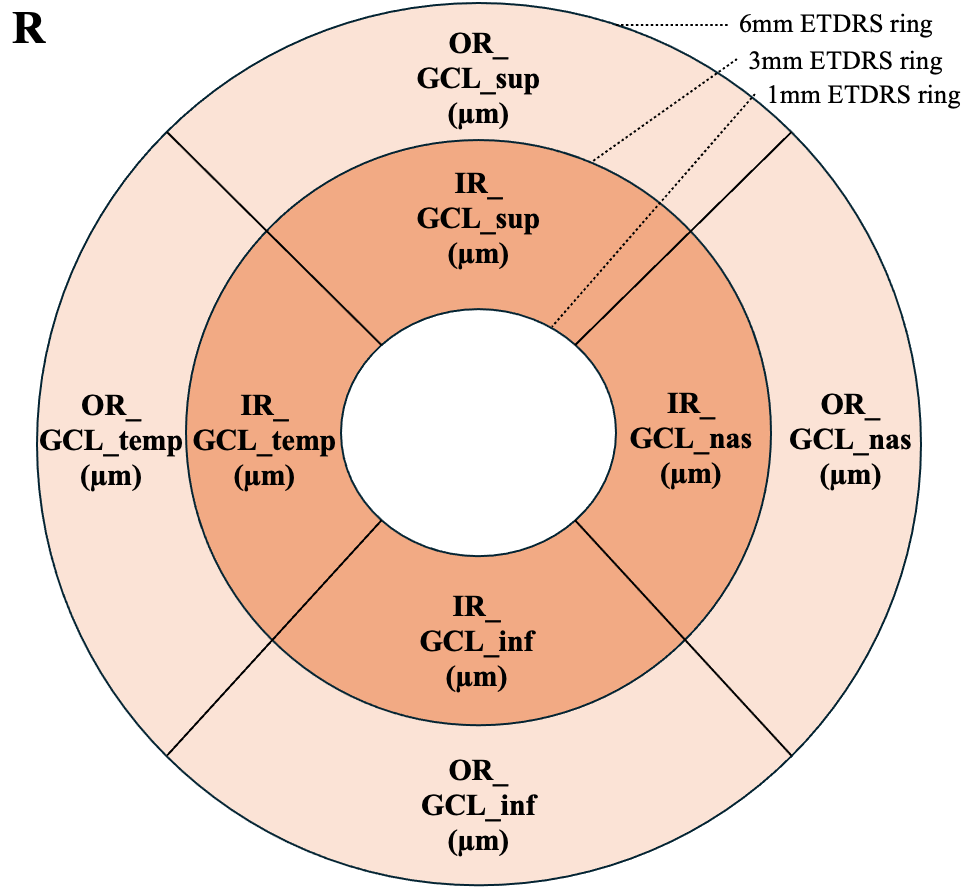 |
| --- | --- |
| **Average IPL thickness:**  ***average thickness inner ring (avT_IPL_IR)***  = (IR_IPL_sup + IR_IPL_nas + IR_IPL_inf + IR_IPL_temp)/4  ***average thickness outer ring (avT_IPL_OR)***  = (OR_IPL_sup + OR_IPL_nas + OR_IPL_inf + OR_IPL_temp)/4  ***avT_IPL_IR+OR = (avT_IPL_IR*2 + avT_IPL_OR*6,75)/8,75*** | 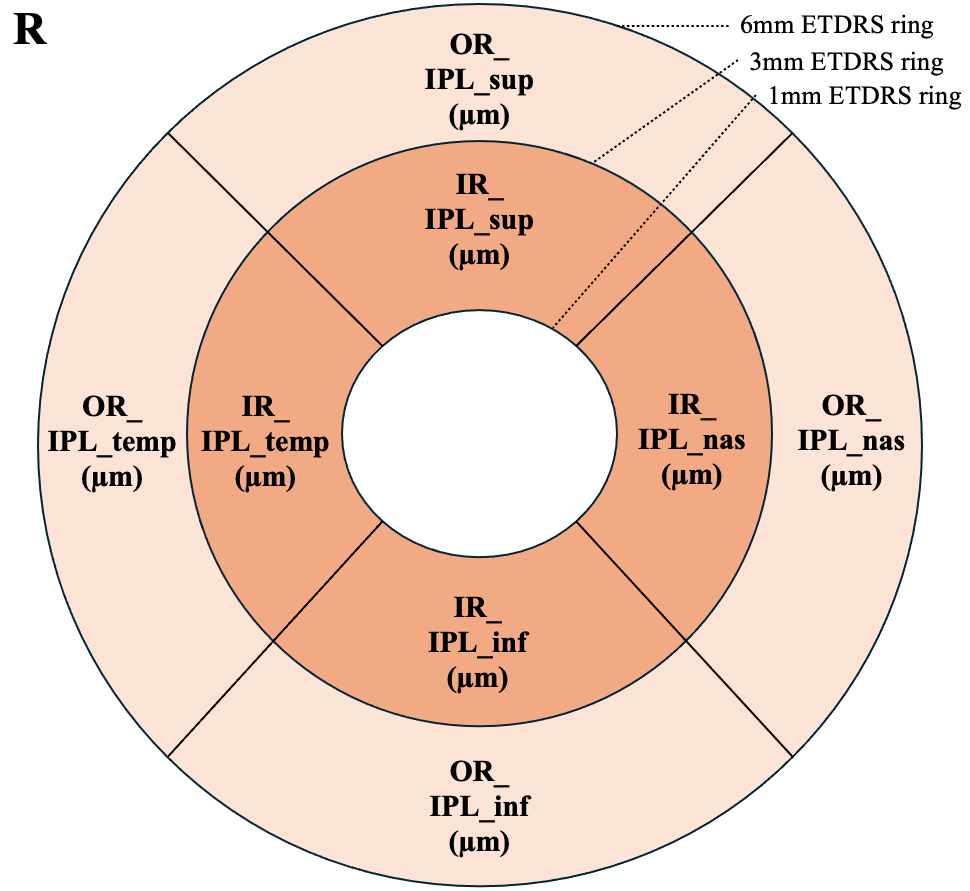 |
| **GCIPL thickness = average GCL thickness + average IPL thickness**  **avT_GCL_IR+OR + avT_IPL_IR+OR** | |
| avT: average thickness  IR: inner ETDRS ring (1-3mm) OR: outer ETDRS ring (3-6mm) | |

In accordance with recommendations for use of optical coherence tomography within McDonald 2024 criteria (see reference 3).
