# Supplementary material for: Application of optical coherence tomography in multiple sclerosis: consensus recommendations of the Austrian network (AN-OCT-MS)
Source: J Neurol. 2025 Dec 13;273(1):24. doi: 10.1007/s00415-025-13537-8 (PMC12701873; doi:10.1007/s00415-025-13537-8)
Supplement: Supplementary file 2 — Supplementary file2 (DOCX 15 KB) [file 415_2025_13537_MOESM2_ESM.docx]

**Box 2: Clinical examples of the added value of OCT in multiple sclerosis.**

| ***Case 1:*** Female, 26 years old, previously completely healthy.  Initial symptom: Subacute internuclear ophthalmoplegia (INO) on the right side  Cerebral MRI: Detection of a single T2 hyperintense lesion pontine on the right with gadolinium uptake  Spinal MRI: unremarkable  Lumbar puncture: 17 cells/µl, oligoclonal bands positive, otherwise unremarkable  Diagnosis according to McDonald criteria 2017: Dissemination in space: not fulfilled (1/4)  Dissemination in time: fulfilled  **Demyelinating event of unclear significance**  OCT: pRNFL global right: 87µm left: 98µm IED: 11µm  GCIPL global right: 71µm left: 81µm IED: 10µm  *Signs of subclinical optic neuritis on the right*  No signs of advanced MS-associated neuroaxonal damage independent of subclinical right-sided optic neuritis  No signs of other pathology  Diagnosis according to McDonald criteria 2024: *Dissemination in space: fulfilled (2/5)*  Dissemination in time: fulfilled  **Relapsing multiple sclerosis (RMS)**  Initiation of DMT |
| --- |
| **Case 2:** Male, 39 years, migraine with aura  Initial symptom: Subacute incomplete sensory cross-section TH6  Episode about 3 years ago with blurred vision in the left eye, interpreted as migraine at the time  Cerebral MRI: Two single non-specific T2 hyperintense deep-white matter lesions in the frontal right and parietal left  Spinal MRI: T2 hyperintense lesion at the level of C5-6 with gadolinium uptake  Lumbar puncture: 54 cells/µl, oligoclonal bands positive, otherwise unremarkable  Diagnosis according to McDonald criteria 2017: Dissemination in space: not fulfilled (1/4)  Dissemination in time: fulfilled  **Demyelinating event of unclear significance**  OCT: pRNFL global right: 86µm left: 78µm IED: 8µm  GCIPL global right: 74µm left: 65µm IED: 9µm  *Signs of past optic neuritis on the left*  *Signs of advanced MS-associated neuroaxonal damage independent of past left-sided optic neuritis*  No signs of other pathology  Diagnosis according to McDonald criteria 2024: *Dissemination in space: fulfilled (2/5)*  Dissemination in time: fulfilled  **Relapsing multiple sclerosis (RMS)**  *Initiation of highly-effective DMT* |
