# Supplementary material for: Application of optical coherence tomography in multiple sclerosis: consensus recommendations of the Austrian network (AN-OCT-MS)
Source: J Neurol. 2025 Dec 13;273(1):24. doi: 10.1007/s00415-025-13537-8 (PMC12701873; doi:10.1007/s00415-025-13537-8)
Supplement: Supplementary file 3 — Supplementary file3 (DOCX 17 KB) [file 415_2025_13537_MOESM3_ESM.docx]

**Consensus recommendations**

***Indications***

OCT should be performed according to the AN-OCT-MS protocol as part of the diagnostic workup for suspected MS. (strong consensus [100%, 12 votes for, 0 against, 0 abstained],

If a pathological IED is detected in the pRNFL (IED ≥6µm/≥6%) and/or in the GCIPL (IED ≥4µm/≥4%), involvement of the optic nerve (for fulfillment of DIS) is considered proven, provided that other causes have been excluded. (strong consensus [100%], 12 votes for, 0 against, 0 abstained)

OCT according to the AN-OCT-MS protocol should be performed at the time of diagnosis or before treatment is adjusted or changed. (strong consensus [100%], , 12 votes for, 0 against, 0 abstained)

If there are signs of advanced neuroaxonal damage (pRNFL ≤88µm and/or GCIPL<77µm) on OCT, an increased risk of disability progression over the next 3 years should be assumed, provided other causes have been excluded. (strong consensus [100%], 12 votes for, 0 against, 0 abstained)

OCT according to AN-OCT-MS protocol should be performed at the earliest 3 months after any optic neuritis. (strong consensus [100%], 12 votes for, 0 against, 0 abstained)

If a pRNFL loss of >20µm or a GCIPL loss of >10µm is detected in the affected eye after optic neuritis, a significant increase in the risk of incomplete regression of ON and possibly also future relapses can be assumed, provided that other causes have been excluded. (strong consensus [90%], 9 votes for, 1 against, 2 abstained)

The use of OCT in DMT monitoring cannot currently be generally recommended. At centers particularly familiar with the use of OCT, it can be used to quantify MS-associated neuroaxonal damage in the longitudinal section, whereby the interpretation should only be made in the overall clinical context and should never be used as the sole decision criterion. (strong consensus [100%], 11 votes for, 0 against, 1 abstained)

***Quality standard***

Neurologists specializing in MS should create a defined local or regional network in interdisciplinary exchange with intra- or extramural ophthalmologists, taking into account the personnel and local conditions, in order to make OCT according to the AN-OCT-MS protocol accessible to MS patients. (strong consensus [100%], 12 votes for, 0 against, 0 abstained)

The referral of MS patients for OCT should only be made by neurologists specializing in MS centers and should contain at least the following information: Diagnosis, clinical evidence of acute or past optic neuritis, indication(s) (AN-OCT-MS protocol in indication 1/2/3/4). (strong consensus [100%], 12 votes for, 0 against, 0 abstained)

OCT scans in MS patients should include a peripapillary ring scan and a multilinear macular scan according to the AN-OCT-MS protocol. (strong consensus [100%], 12 votes for, 0 against, 0 abstained)

OCT scans in MS patients should undergo quality control based on the OSCAR-IB criteria and only be used for reporting if the quality criteria are met. (strong consensus [100%], 12 votes for, 0 against, 0 abstained)

Sevice platforms Spectralis® (Heidelberg Engineering) or Cirrus® (Carl Zeiss Meditec) should be used for OCT scans in MS patients, as other device platforms are not currently validated for use in MS (strong consensus [100%], 9 votes for, 0 against, 3 abstained)

Longitudinal follow-up OCT scans should be performed with the same device and the follow-up function activated. (strong consensus [100%], 12 votes for, 0 against, 0 abstained)

OCT scans in MS patients should only be performed by physicians/technicians who are familiar with the assessment of the fundus and OCT imaging. (strong consensus [100%], 12 votes for, 0 against, 0 abstained)

Reports of OCT findings according to the AN-OCT-MS protocol should meet the quality criteria of validity, reliability/reproducibility, completeness, comprehensibility, comparability, objectivity and usefulness. (strong consensus [100%], 12 votes for, 0 against, 0 abstained)

Reports of OCTs according to the AN-OCT-MS protocol should in any case indicate which device was used to acquire the OCT scan and whether the OCT scan meets the OSCAR-IB quality criteria. (strong consensus [100%], 12 votes for, 0 against, 0 abstained)

The findings of an OCT according to the AN-OCT-MS protocol should be accompanied by the underlying OCT scan. Possible formats for this are a paper printout, a PDF file or a DICOM file. (strong consensus [100%], 12 votes for, 0 against, 0 abstained)

The findings of an OCT according to the AN-OCT-MS protocol should explicitly and clearly show the relevant measured variables (global layer thicknesses of pRNFL and GCIPL in the right and left eye) as numerical values. (strong consensus [100%], 12 votes for, 0 against, 0 abstained)

The findings of an OCT according to the AN-OCT-MS protocol should contain a summarizing interpretation that explicitly includes the following points:

- Is there evidence of secondary pathology independent of MS that affects the interpretation with respect to indications 1-4?
- Are there signs of optic neuritis according to indication 1?
- Are there signs of advanced MS-associated neuroaxonal damage according to indication 2?
- In case of referral after optic neuritis (indication 3): What is the extent of neuroaxonal loss in the affected eye (incl. indication of loss in pRNFL and GCIPL in µm)

(strong consensus [100%], 12 votes for, 0 against, 0 abstained)
